# Development and validation of a machine learning model for predicting early postoperative complications after radical gastrectomy

**DOI:** 10.3389/fonc.2025.1631260

**Published:** 2025-10-14

**Authors:** Ruyin Li, Zirui Zhao, Jianchun Yu

**Affiliations:** ^1^ Department of General Surgery, Peking Union Medical College Hospital, Peking Union Medical College and Chinese Academy of Medical Sciences, Beijing, China; ^2^ Department of Neurology, Peking Union Medical College Hospital, Peking Union Medical College and Chinese Academy of Medical Sciences, Beijing, China

**Keywords:** gastric cancer, complication, machine learning, predictive model, postoperative outcome

## Abstract

**Background:**

Postoperative complications significantly impact gastric cancer patients’ recovery and remain a major research focus. This study aimed to develop a machine learning model utilizing preoperative and intraoperative data to stratify the risk of early postoperative complications in patients undergoing radical gastrectomy.

**Methods:**

Clinical data from gastric cancer patients who underwent radical gastrectomy at Peking Union Medical College Hospital between 2014 and 2024 were retrospectively collected. Using R software, ten machine learning algorithms—including eXtreme Gradient Boosting, Support Vector Machine, random forest, Neural Network, naive Bayes, logistic regression, Linear Discriminant Analysis, K-Nearest Neighbors, Generalized Linear Model with Elastic-Net Regularization and classification tree—were employed to construct predictive models for early postoperative complications. Nested cross-validation was applied for model validation, and performance was evaluated using receiver operating characteristic curves, decision curve analysis, and calibration curves.

**Results:**

A total of 926 patients were included in this study, comprising 667 males (72%) and 259 females (28%), with 131 (14.13%) suffering postoperative complications. Predictive features included smoking, Nutritional Risk Screening 2002 score>3, reconstruction, clinical T-stage>1, operative time, neoadjuvant chemotherapy combined with immunotherapy or targeted therapy, and resection site. Among the ten models, eXtreme Gradient Boosting demonstrated the best predictive performance, achieving an area under the receiver operating characteristic curve (AUC) of 0.788, along with superior calibration and decision curve analysis results.

**Conclusion:**

Based on preoperative and intraoperative data, the eXtreme Gradient Boosting model demonstrated the strongest predictive capability for postoperative complications following radical gastrectomy.These findings underscore the potential of machine learning-based models in stratifying the risk of early postoperative complications in patients undergoing radical gastrectomy, thereby enhancing clinical decision-making and improving patient outcomes in gastric cancer surgery.

## Introduction

Gastric cancer (GC) is a common malignant tumor of the digestive system, ranking as the fifth most prevalent cancer globally and the third leading cause of cancer-related mortality (1, 2). Currently, surgical resection remains the most reliable and mainstream treatment for GC, with postoperative recovery playing a critical role in patient prognosis (3–6). Among the factors influencing patient recovery, short-term postoperative outcomes, particularly the occurrence of complications, hold significant clinical importance.

Machine learning (ML) has emerged as a cutting-edge tool in oncological research, offering substantial advantages over traditional statistical methods in constructing diagnostic and prognostic models (7, 8). In cancer studies, algorithms such as eXtreme Gradient Boosting (XGBoost), SVM (Support Vector Machine), random forest, Neural Network (NNET), naive Bayes, logistic regression, Linear Discriminant Analysis (LDA), K-Nearest Neighbors (KNN), Generalized Linear Model with Elastic-Net Regularization (GLMNet), and classification trees are widely employed for predictive modeling (9), with analysis typically conducted using R or Python.

Recent years have seen extensive research on postoperative complications in gastric cancer (10, 11).Several studies have applied ML to develop predictive models for such complication (6, 12, 13),however, many of these excluded patients who underwent neoadjuvant therapy or included only minimal data from this subgroup. To address this gap, our study using preoperative as well as intraoperative data incorporated a substantial cohort of neoadjuvant therapy recipients and developed ML-based predictive models to stratify the risk of early postoperative complications in GC. We anticipate that these models will facilitate clinical decision-making, assist in complication prevention, and ultimately promote accelerated postoperative recovery.

## Materials and method

### Patients selection

We retrospectively collected data from patients who underwent radical gastrectomy for GC at Peking Union Medical College Hospital between 2014 and 2024. Inclusion criteria were:(1) undergoing radical gastrectomy; (2) histopathological confirmation of gastric adenocarcinoma; (3) surgery performed by experienced gastrointestinal surgeons. Exclusion criteria included: (1) Age <18 or >80 years; (2) intraoperative detection of metastatic disease or other evidence of metastasis; (3) concurrent other malignant tumors; (4) severe cardiovascular, cerebrovascular, or other systemic comorbidities; (5) missing data exceeding 30% or loss to follow-up.

Definition of complications: Early postoperative complications were defined as any Clavien-Dindo grade ≥2 events occurring within 30 days after surgery.

### Data collection

We collected demographic and clinical data from enrolled patients while controlling for potential confounding variables using the Directed Acyclic Graph (DGA) principle. Demographic characteristics included age and sex. Cinical data included preoperative data, intraoperative data and postoperative outcome. Preoperative clinical data encompassed length of preoperative hospitalization, smoking and alcohol consumption history, comorbidities (hypertension, diabetes mellitus, reflux esophagitis, pyloric obstruction), psychological disorders, previous abdominal surgery, H. pylori(HP) infection status, family history of malignant tumors, neoadjuvant treatment therapy, Nutritional Risk Screening-2002 (NRS-2002) score, and laboratory parameters including white blood cell(WBC) count, hemoglobin, glucose, albumin, albumin-to-globulin ratio(A/G), C-reactive protein(CRP), D-dimer, and tumor markers (CA242, AFP, CEA, CA19-9, CA724). Tumor location, clinical TNM stage (according to the AJCC 8th edition criteria), and Her-2 expression status were determined based on preoperative imaging and endoscopic biopsy findings. Intraoperative data consisted of resection site, anastomosis method, operative duration, intraoperative blood loss, endoscopy utilization, feeding tube placement, and blood transfusion. The primary postoperative outcome was the occurrence of complications.

### Data analysis strategy

For variables with missing values less than 30%, multiple imputation was applied to the collected data. Univariate analysis and LASSO regression were performed on the processed data. In the univariate analysis, categorical data were assessed using the chi-square test or Fisher’s exact test, normally distributed numerical data were analyzed using the t-test (results presented as mean ± standard deviation), and non-normally distributed numerical data were evaluated using the rank-sum test (results expressed as median [25%;75%]. A p-value less than 0.05 was considered statistically significant. In the LASSO analysis, the optimal regularization parameter (λ) was selected, and all factors with non-zero coefficients were extracted. The intersection of these factors was used to construct ML models.

Based on the mlr3 system and relevant R packages, the following models were built and tuned: XGBoost, SVM, Random Forest, NNET, Naive Bayes, Logistic Regression, LDA, KNN, GLMNet, and Classification Tree. Nested cross-validation (Outer 5-fold CV +Inner 5-fold CV, resolution =3) was employed to evaluate these models, yielding AUC, accuracy, recall, and specificity. Receiver operating characteristic (ROC) curves, decision curve analysis (DCA) curves, and calibration curves were plotted to assess model performance, and the best-performing predictive model was selected.

## Results

A total of 926 patients who underwent GC surgery were included in this study, comprising 667 males (72%) and 259 females (28%), with a median age of 61 years [53;67]. The baseline demographic and clinical characteristics are presented in **Table 1**. Postoperative complications occurred in 131 patients (14.13%), including anastomotic leakage, gastroparesis, hemorrhage, infection, obstruction, acute cardiovascular and cerebrovascular events.

**Table 1:** Baseline characteristics and univariate analysis.

Variables	[ALL]	No	Yes	Univariate analysis		
	*N=926*	*N=795*	*N=131*	OR	P.ratio	P.overall
age	61.0 [53.0;67.0]	61.0 [53.0;67.0]	63.0 [56.0;68.0]	1.02 [1.00;1.04]	0.044	0.040
sex:						0.019
female	259 (28.0%)	234 (29.4%)	25 (19.1%)	Ref.	Ref.	
male	667 (72.0%)	561 (70.6%)	106 (80.9%)	1.76 [1.13;2.85]	0.012	
preoperative hospitalization	5.00 [4.00;7.00]	5.00 [4.00;7.00]	6.00 [4.00;8.00]	1.04 [0.99;1.08]	0.119	0.010
smoking history:						0.001
no	492 (53.1%)	441 (55.5%)	51 (38.9%)	Ref.	Ref.	
yes	434 (46.9%)	354 (44.5%)	80 (61.1%)	1.95 [1.34;2.86]	<0.001	
drinking history:						0.677
no	640 (69.1%)	552 (69.4%)	88 (67.2%)	Ref.	Ref.	
yes	286 (30.9%)	243 (30.6%)	43 (32.8%)	1.11 [0.74;1.64]	0.601	
hypertension:						0.447
no	624 (67.4%)	540 (67.9%)	84 (64.1%)	Ref.	Ref.	
yes	302 (32.6%)	255 (32.1%)	47 (35.9%)	1.19 [0.80;1.74]	0.391	
diabetes:						0.532
no	750 (81.0%)	647 (81.4%)	103 (78.6%)	Ref.	Ref.	
yes	176 (19.0%)	148 (18.6%)	28 (21.4%)	1.19 [0.74;1.86]	0.455	
reflux esophagitis:						1.000
no	883 (95.4%)	758 (95.3%)	125 (95.4%)	Ref.	Ref.	
yes	43 (4.64%)	37 (4.65%)	6 (4.58%)	1.00 [0.37;2.27]	0.992	
pyloric obstruction:						0.593
no	888 (95.9%)	764 (96.1%)	124 (94.7%)	Ref.	Ref.	
yes	38 (4.10%)	31 (3.90%)	7 (5.34%)	1.41 [0.56;3.12]	0.439	
psychological disorder:						0.316
no	918 (99.1%)	789 (99.2%)	129 (98.5%)	Ref.	Ref.	
yes	8 (0.86%)	6 (0.75%)	2 (1.53%)	2.14 [0.28;9.74]	0.406	
abdominopelvic surgery history:						0.862
no	712 (76.9%)	610 (76.7%)	102 (77.9%)	Ref.	Ref.	
yes	214 (23.1%)	185 (23.3%)	29 (22.1%)	0.94 [0.59;1.45]	0.787	
ESD history:						0.755
no	906 (97.8%)	778 (97.9%)	128 (97.7%)	Ref.	Ref.	
yes	20 (2.16%)	17 (2.14%)	3 (2.29%)	1.12 [0.25;3.42]	0.865	
HP infection:						0.780
no	795 (85.9%)	681 (85.7%)	114 (87.0%)	Ref.	Ref.	
yes	131 (14.1%)	114 (14.3%)	17 (13.0%)	0.90 [0.50;1.52]	0.695	
family history of tumor:						0.690
no	702 (75.8%)	605 (76.1%)	97 (74.0%)	Ref.	Ref.	
yes	224 (24.2%)	190 (23.9%)	34 (26.0%)	1.12 [0.72;1.69]	0.606	
BMI	23.7 [21.7;26.0]	23.8 [21.6;26.0]	23.7 [22.0;26.1]	1.00 [0.95;1.06]	0.913	0.702
neoadjuvant chemotherapy combined with immunotherapy or target therapy:						0.015
no	878 (94.8%)	760 (95.6%)	118 (90.1%)	Ref.	Ref.	
yes	48 (5.18%)	35 (4.40%)	13 (9.92%)	2.41 [1.19;4.59]	0.016	
NRS2002 score>3:						0.006
no	841 (90.8%)	731 (91.9%)	110 (84.0%)	Ref.	Ref.	
yes	85 (9.18%)	64 (8.05%)	21 (16.0%)	2.19 [1.26;3.68]	0.006	
WBC:						0.633
high	19 (2.05%)	15 (1.89%)	4 (3.05%)	Ref.	Ref.	
low	79 (8.53%)	68 (8.55%)	11 (8.40%)	0.60 [0.17;2.48]	0.453	
normal	828 (89.4%)	712 (89.6%)	116 (88.5%)	0.59 [0.21;2.18]	0.395	
HGB:						0.671
high	62 (6.70%)	51 (6.42%)	11 (8.40%)	Ref.	Ref.	
low	150 (16.2%)	128 (16.1%)	22 (16.8%)	0.79 [0.36;1.82]	0.574	
normal	714 (77.1%)	616 (77.5%)	98 (74.8%)	0.73 [0.38;1.53]	0.385	
albumin:						0.579
low	28 (3.02%)	23 (2.89%)	5 (3.82%)	Ref.	Ref.	
normal	898 (97.0%)	772 (97.1%)	126 (96.2%)	0.73 [0.29;2.26]	0.555	
albumin-to-globulin ratio:						0.195
high	6 (0.65%)	5 (0.63%)	1 (0.76%)	Ref.	Ref.	
low	2 (0.22%)	1 (0.13%)	1 (0.76%)	3.87 [0.07;229]	0.500	
normal	918 (99.1%)	789 (99.2%)	129 (98.5%)	0.74 [0.11;19.6]	0.800	
G:						0.333
high	177 (19.1%)	146 (18.4%)	31 (23.7%)	Ref.	Ref.	
low	9 (0.97%)	8 (1.01%)	1 (0.76%)	0.66 [0.03;3.88]	0.697	
normal	740 (79.9%)	641 (80.6%)	99 (75.6%)	0.73 [0.47;1.14]	0.164	
CRP:	1.04 [0.50;2.23]					0.203
high	178 (19.2%)	147 (18.5%)	31 (23.7%)	Ref.	Ref.	
normal	748 (80.8%)	648 (81.5%)	100 (76.3%)	0.73 [0.47;1.15]	0.171	
Di-dimer:						0.169
high	241 (26.0%)	200 (25.2%)	41 (31.3%)	Ref.	Ref.	
normal	685 (74.0%)	595 (74.8%)	90 (68.7%)	0.74 [0.50;1.11]	0.143	
CA242	6.60 [3.42;11.5]	6.60 [3.35;11.5]	6.60 [3.85;12.1]	1.00 [1.00;1.01]	0.103	0.647
AFP	2.70 [2.00;4.10]	2.70 [2.00;4.10]	2.70 [2.00;3.70]	0.97 [0.93;1.02]	0.290	0.403
CEA	2.20 [1.40;3.32]	2.11 [1.40;3.30]	2.28 [1.50;3.41]	1.00 [0.98;1.01]	0.740	0.421
CA199	10.1 [6.43;16.7]	9.90 [6.40;16.8]	11.3 [6.70;16.5]	1.00 [1.00;1.00]	0.611	0.395
CA724	2.30 [1.50;6.00]	2.40 [1.50;6.00]	2.00 [1.50;5.65]	0.99 [0.98;1.01]	0.343	0.225
excision extent:						0.043
distal	572 (61.8%)	503 (63.3%)	69 (52.7%)	Ref.	Ref.	
proximal	40 (4.32%)	35 (4.40%)	5 (3.82%)	1.07 [0.35;2.60]	0.896	
total	314 (33.9%)	257 (32.3%)	57 (43.5%)	1.62 [1.10;2.37]	0.015	
reconstruction:						<0.001
billroth1	432 (46.7%)	404 (50.8%)	28 (21.4%)	Ref.	Ref.	
billroth2	12 (1.30%)	9 (1.13%)	3 (2.29%)	4.93 [0.99;18.0]	0.051	
oesophago-gastric	40 (4.32%)	35 (4.40%)	5 (3.82%)	2.10 [0.67;5.42]	0.186	
Roux-en-y	442 (47.7%)	347 (43.6%)	95 (72.5%)	3.93 [2.55;6.24]	<0.001	
intraoperative transfusion:						0.128
no	892 (96.3%)	769 (96.7%)	123 (93.9%)	Ref.	Ref.	
yes	34 (3.67%)	26 (3.27%)	8 (6.11%)	1.95 [0.80;4.24]	0.134	
intraoperative gastroscopy:						1.000
no	875 (94.5%)	751 (94.5%)	124 (94.7%)	Ref.	Ref.	
yes	51 (5.51%)	44 (5.53%)	7 (5.34%)	0.98 [0.39;2.10]	0.964	
blood loss≥50:						0.001
no	663 (71.6%)	586 (73.7%)	77 (58.8%)	Ref.	Ref.	
yes	263 (28.4%)	209 (26.3%)	54 (41.2%)	1.97 [1.34;2.88]	0.001	
operative duration	3.65 [3.10;4.50]	3.50 [3.00;4.25]	4.50 [3.78;5.15]	2.15 [1.79;2.58]	<0.001	<0.001
location(preoperative):						0.093
antrum	316 (34.1%)	283 (35.6%)	33 (25.2%)	Ref.	Ref.	
body	190 (20.5%)	160 (20.1%)	30 (22.9%)	1.61 [0.94;2.74]	0.083	
diffuse	159 (17.2%)	135 (17.0%)	24 (18.3%)	1.53 [0.86;2.68]	0.148	
GEJ	147 (15.9%)	118 (14.8%)	29 (22.1%)	2.10 [1.22;3.63]	0.008	
incisura angularis	114 (12.3%)	99 (12.5%)	15 (11.5%)	1.31 [0.66;2.47]	0.432	
Her2(biopsy):						0.507
no	396 (42.8%)	336 (42.3%)	60 (45.8%)	Ref.	Ref.	
yes	530 (57.2%)	459 (57.7%)	71 (54.2%)	0.87 [0.60;1.26]	0.450	
cT-stage>1:						0.003
no	305 (32.9%)	277 (34.8%)	28 (21.4%)	Ref.	Ref.	
yes	621 (67.1%)	518 (65.2%)	103 (78.6%)	1.96 [1.27;3.10]	0.002	
cN-stage:						0.076
no	487 (52.6%)	428 (53.8%)	59 (45.0%)	Ref.	Ref.	
yes	439 (47.4%)	367 (46.2%)	72 (55.0%)	1.42 [0.98;2.07]	0.063	

The univariate analysis results are presented in **Table 1**, which shows significant differences between the complication and non-complication groups in the following variables: age(p=0.04), sex(p=0.019), preoperative hospitalization(p=0.01), smoking history(p=0.001), neoadjuvant chemotherapy combined with immunotherapy or targeted therapy(p=0.015), NRS2002 score>3(p=0.006), resection extent(p=0.043), anastomosis method(p<0.001), blood loss ≥50 mL(p=0.001), operative time(p<0.001), clinical T(cT)-stage>1(p=0.003).

**Figure 1:**
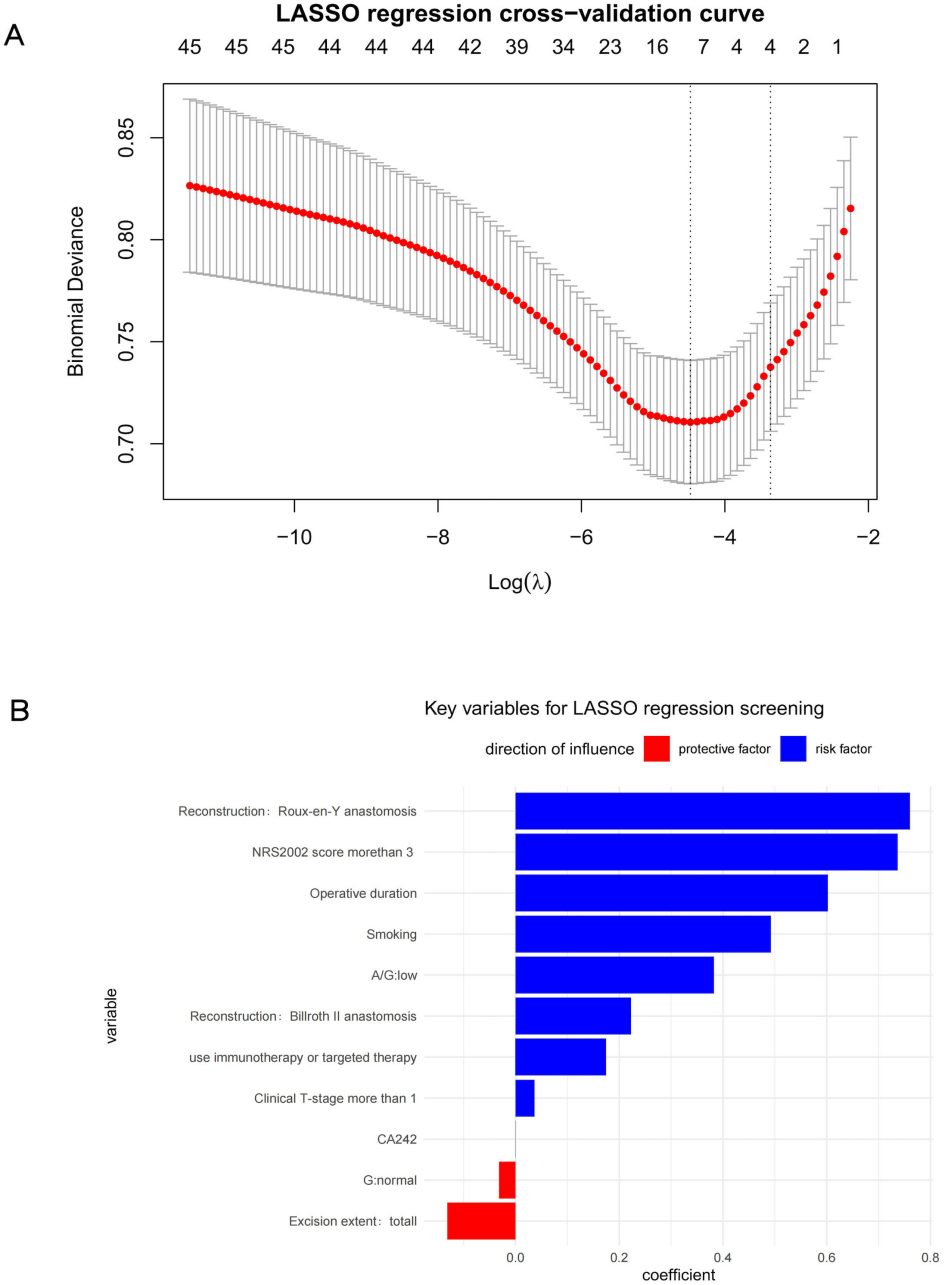
**(A)** LASSO regression cross−validation curve **(B)** Key variables for LASSO regression screening.

The LASSO regression results are illustrated in **Figure 1**, revealing that factors significantly associated with postoperative complications included Roux-en-Y anastomosis, NRS2002 score >3, prolonged operative time, smoking, A/G, Billrouth II anastomosis, neoadjuvant therapy combined with immunotherapy or targeted therapy, cT-stage>1, G and excition extent. Weakly correlated factors included CA242.

Based on the combined results of univariate and LASSO analyses, the following variables were selected for ML model construction: smoking, NRS2002 score >3, reconstruction method, cT-stage >1, operative time, neoadjuvant chemotherapy combined with immunotherapy or targeted therapy and resection extent.

**Figure 2:**
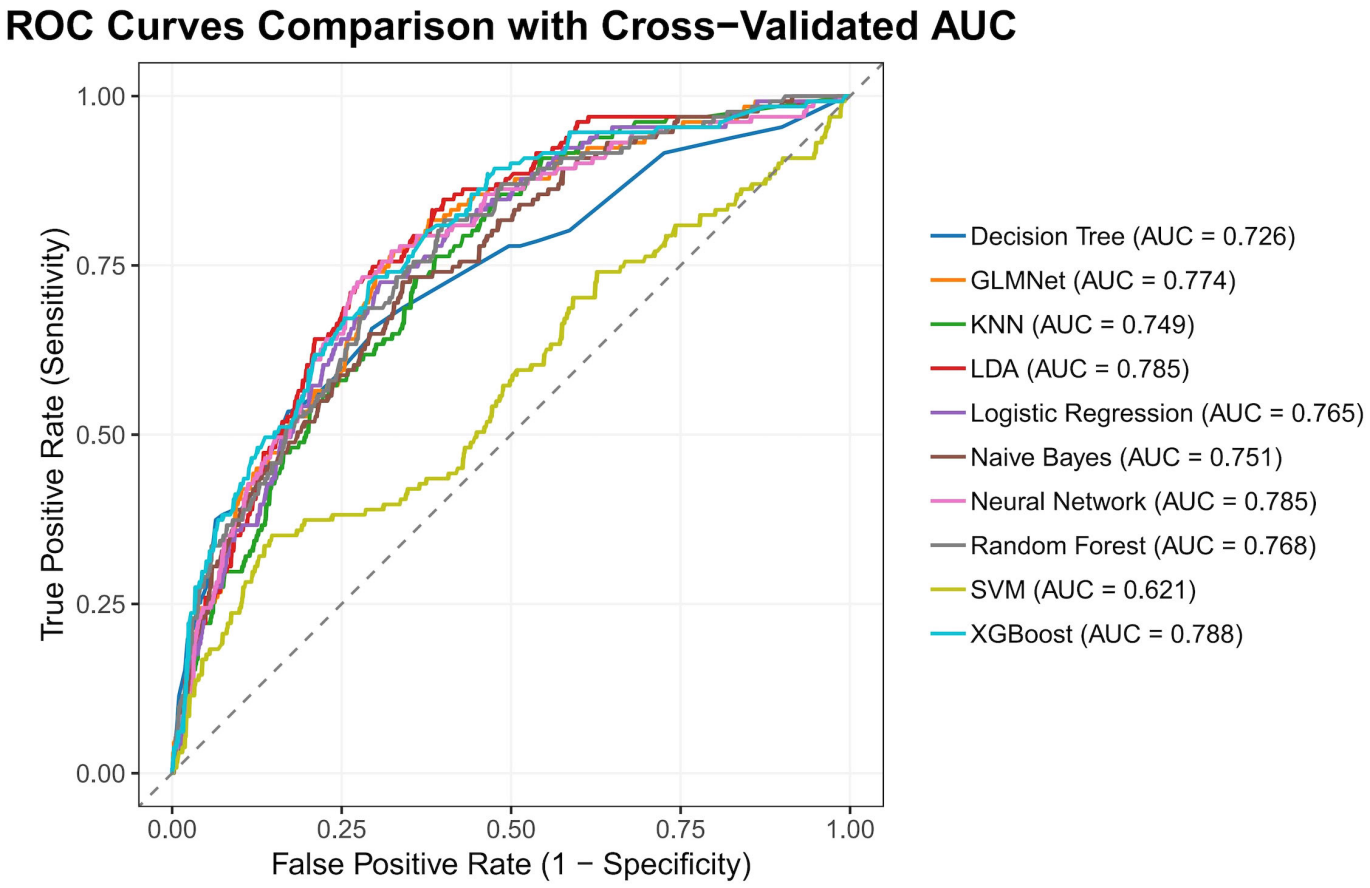
Receiver operating characteristic curves of the models.

**Figure 3:**
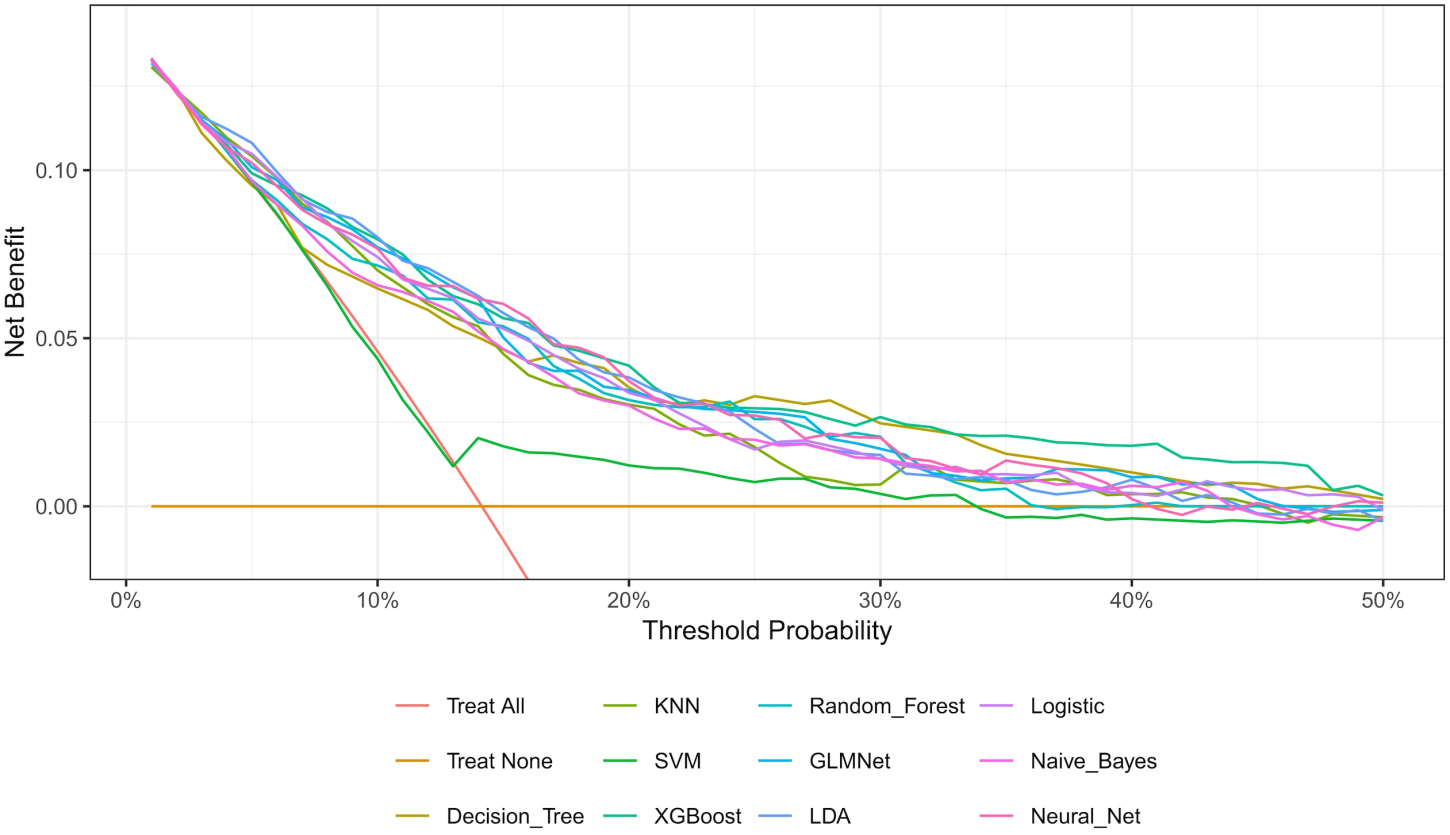
Decision curve analysis (DCA) of the models.

**Figure 4:**
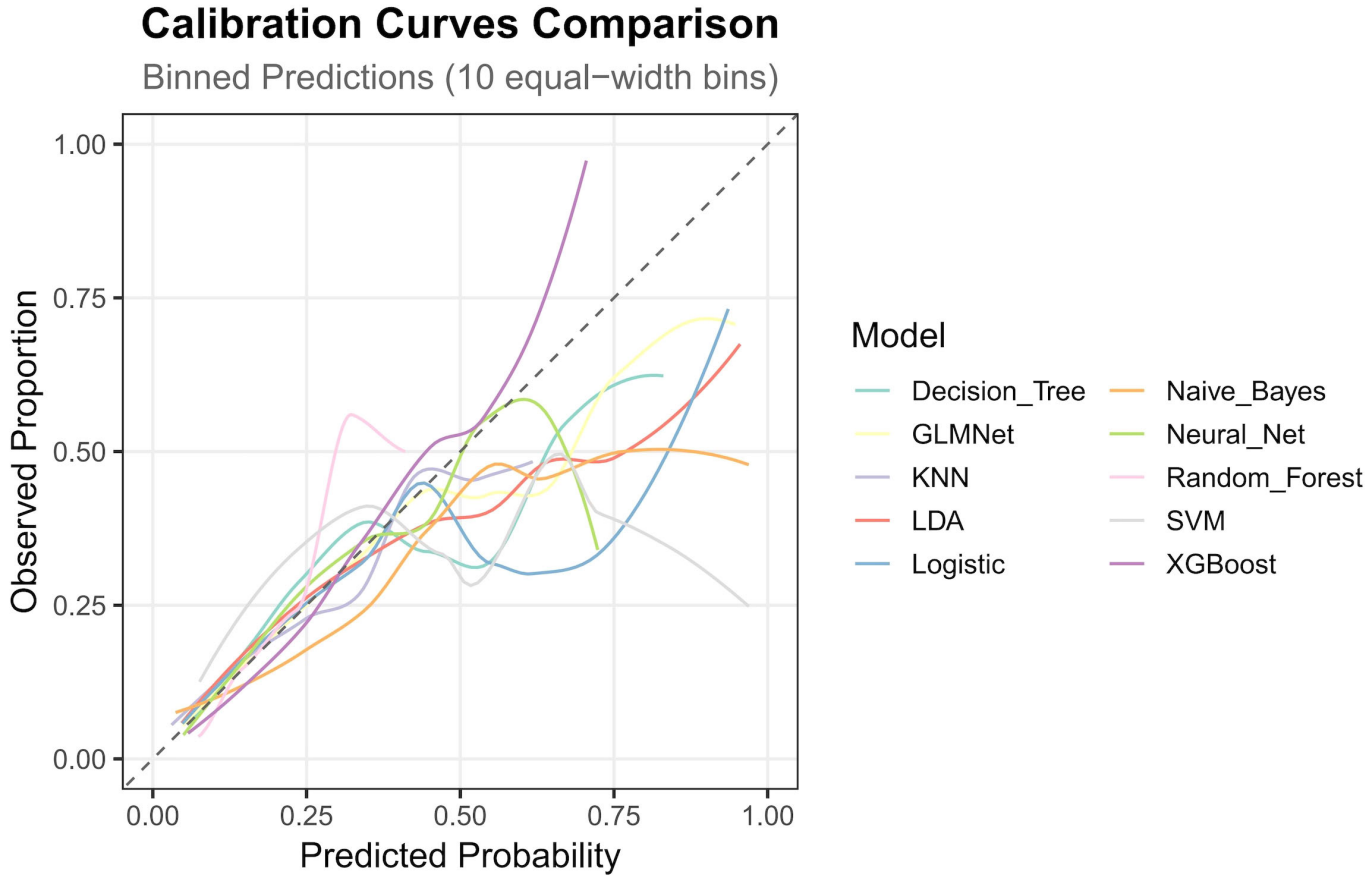
The calibration curves of the models.

The nested cross-validation results for the XGBoost, SVM, Random Forest, NNET, Naive Bayes, Logistic Regression, LDA, KNN, GLMNet, and Classification Tree models are presented in **Table 2**. Among these models, XGBoost demonstrated the highest area under the receiver operating characteristic curve (AUC = 0.788) and a comparatively high recall value (0.741), indicating superior predictive performance. The ROC curves for each model are presented in **Figure 2**. The DCA and calibration curves for each model are shown in **Figures 3** and **4**. In the DCA, XGBoost provided the highest clinical net benefit, confirming its status as one of the top-performing models. Additionally, the calibration curve indicated that XGBoost had the best agreement between predicted and observed probabilities. In conclusion, the XGBoost model exhibited optimal performance in predicting postoperative complications in GC patients.

**Table 2:** Performance metrics of the models.

Model	AUC [95%CI]	Accuracy [95%CI]	Recall [95%CI]	Specificity [95%]
Decision Tree	0.726 [0.645, 0.808]	0.725 [0.694, 0.755]	0.606 [0.464, 0.748]	0.745 [0.692, 0.797]
KNN	0.749 [0.660, 0.838]	0.662 [0.620, 0.703]	0.681 [0.542, 0.821]	0.657 [0.595, 0.720]
SVM	0.621 [0.543, 0.700]	0.819 [0.784, 0.853]	0.215 [0.076, 0.354]	0.918 [0.867, 0.969]
XGBoost	0.788 [0.746, 0.830]	0.687 [0.644, 0.729]	0.741 [0.684, 0.799]	0.678 [0.619, 0.736]
Random Forest	0.768 [0.708, 0.827]	0.678 [0.603, 0.753]	0.732 [0.679, 0.785]	0.67 [0.584, 0.756]
GLMNet	0.774 [0.724, 0.824]	0.715 [0.671, 0.759]	0.648 [0.563, 0.733]	0.727 [0.672, 0.781]
LDA	0.785 [0.746, 0.825]	0.741 [0.713, 0.768]	0.674 [0.571, 0.777]	0.752 [0.711, 0.793]
Logistic	0.765 [0.702, 0.829]	0.725 [0.675, 0.774]	0.653 [0.501, 0.805]	0.736 [0.669, 0.804]
Naïve Bayes	0.751 [0.694, 0.808]	0.68 [0.623, 0.737]	0.669 [0.497, 0.842]	0.682 [0.599, 0.764]
Neural Net	0.785 [0.691, 0.880]	0.716 [0.658, 0.774]	0.734 [0.573, 0.895]	0.714 [0.652, 0.777]

## Discussion

Postoperative complications represent the most critical factor affecting recovery in GC patients and have long been a major concern for surgeons. While numerous studies have investigated predictors of various postoperative complications in GC (14, 15),and a limited number of ML-based models have been reported (12, 13),these studies typically excluded patients receiving neoadjuvant therapy. The present study addresses this research gap by including GC patients who underwent neoadjuvant treatment. Furthermore, we developed a machine learning-based predictive model for postoperative complications using preoperative and intraoperative data, which may ultimately guide clinical decision-making to prevent complications and facilitate postoperative recovery.

In terms of methodology, the predictive factors for model construction in this study were selected through a dual approach incorporating both univariate analysis and LASSO regression. This strategy not only preserves statistically significant variables but also accounts for feature interactions, thereby reducing the false-positive rate. Regarding ML algorithms, we employed ten distinct methods—XGBoost, SVM, Random Forest, NNET, Naive Bayes, Logistic Regression, LDA, KNN, GLMNet, and Classification Trees—selected for their suitability given our sample size and outcome characteristics. For model validation, we implemented nested cross-validation, which offers distinct advantages over conventional training-test splits. Specifically, this approach is more appropriate for smaller datasets, rigorously prevents data leakage, and enables phased validation, yielding more robust results (16). As this is a complication-prediction model, the AUC and recall metrics were prioritized in performance evaluation. Additionally, ROC, DCA, and calibration plots were utilized to visually assess the model’s predictive efficacy.

The LASSO and univariate analyses identified, Roux-en-Y anastomosis, NRS2002 score>3, prolonged operative time, smoking, and neoadjuvant chemotherapy combined with immunotherapy or targeted therapy, cT-stage >1 as significant risk factors for postoperative complications, while total gastric resection demonstrated protective effects. These findings suggest patients with smoking history, poor nutritional status, prolonged operative duration, complex surgical approaches, aggressive tumor biology face higher complication risks.

Surgery is the most critical factor contributing to postoperative complications. A multicenter retrospective study involving 2,508 patients (17) demonstrated that Roux-en-Y anastomosis and prolonged operative duration were risk factors for postoperative complications. Smoking and inflammatory status are associated with the development and poor prognosis of various tumors. Research by Kentaro Matsuo et al. (18)found that patients with a history of smoking and higher WBC levels at the gastroesophageal junction were more prone to anastomotic leakage after surgery. The study by Junbo Zuo’s team (19)indicated that malnutrition and sarcopenia were correlated with postoperative complications in GC patients. Luigi Marano (20)revealed that early immunonutrition support for postoperative GC patients significantly reduced the incidence of anastomotic leakage and infection events compared to those without such support. Conversely, Jingxia Lv (21) found that inadequate postoperative nutritional support was a risk factor for poor prognosis after radical gastrectomy. Tumor aggressiveness is also a key factor influencing postoperative complications. Studies have shown that GC patients with neural invasion and higher T-stage have worse prognoses (22, 23). These findings are consistent with our research results, further validating the reliability of this study.

In addition, the results of this study suggest that GC patients receiving neoadjuvant therapy combined with immunotherapy or targeted therapy may be at a higher risk of postoperative complications, which represents a novel finding. In recent years, although numerous studies on neoadjuvant therapy for GC have been published, the majority have focused on comparing the efficacy and safety of chemotherapy combined with targeted/immunotherapeutic agents versus conventional neoadjuvant chemotherapy regimens (24–26). However, there remains a scarcity of comparative analyses regarding postoperative complications between patients who underwent surgery following neoadjuvant therapy incorporating targeted or immunotherapeutic agents and those who did not receive any neoadjuvant treatment. A distinctive feature of this study is the inclusion of both patients who received neoadjuvant therapy and those who did not. It is noteworthy that patients receiving neoadjuvant chemotherapy combined with immunotherapy or targeted therapy often present with features inherently associated with a higher propensity for postoperative complications, such as more advanced tumor stages, broader invasive extent, and more complex surgical procedures.Furthermore, from a mechanistic perspective, previous studies have suggested that immunotherapy and targeted therapy may induce inflammatory responses or immune-related adverse events, thereby impairing tissue healing (27–29). These factors may explain the observed correlation between the combined neoadjuvant immunotherapeutic or targeted regimen and an increased risk of postoperative complications. Nevertheless, the precise mechanisms underlying the elevated risk of postoperative complications associated with neoadjuvant therapy combined with immunotherapy or targeted therapy warrant further investigation.

As shown in **Figures 3** and **4** the XGBoost model demonstrated the best predictive performance among the ML models evaluated. It achieved optimal results across ROC, DCA and calibration curves. Furthermore, **Table 2** shows that the XGBoost model also achieved a high recall, a performance metric that is often more critical than accuracy in predicting complications, especially when dealing with imbalanced class distributions. These findings collectively confirm the high efficiency, accuracy, and clinical utility of the XGBoost prediction model.

During the development of the XGBoost prediction model, both preoperative and intraoperative predictors were incorporated to enable immediate postoperative risk stratification for complications. This approach facilitates early intervention in high-risk patients. Clinically, factors such as smoking history, preoperative NRS2002 score, neoadjuvant therapy regimen, and clinical tumor stage are readily available before surgery. This study has identified smoking history, NRS2002 score >**3**, neoadjuvant therapy combined with targeted or immunotherapy, and cT-stage >1 as significant risk factors for postoperative complications. Therefore, preoperative interventions—including smoking cessation counseling, nutritional support, adequate tumor downstaging, and optimal selection of neoadjuvant therapy—may help mitigate these risks. Furthermore, for patients who are candidates for different surgical approaches, the model can be utilized preoperatively to calculate and compare the predicted risk stratification intervals for complications associated with various surgical techniques, thereby assisting in the selection of the most appropriate procedure. Concurrently, efforts should be made to shorten the operative duration during surgery. Finally, for patients predicted to be at high risk for complications immediately after surgery, more intensive monitoring of their postoperative condition is essential. Timely implementation of corresponding management measures is crucial to accelerate recovery and improve patient prognosis.

The highlight of this study lies in the application of ML methods to establish a predictive model for postoperative complications in all GC patients (including those who underwent neoadjuvant therapy), with the model evaluated using cross-nested validation. Additionally, it was found that chemotherapy combined with immunotherapy or targeted therapy may contribute to the occurrence of postoperative complications. This study also has limitations. It is a single-center retrospective study, which inherently limits the sample size. Furthermore, the inclusion of certain intraoperative variables in this predictive model may partially compromise its utility for preoperative clinical decision-making. In the future, our team will continue to expand the database and incorporate external datasets to further validate the model.

## Conclusion

This study developed a ML-based predictive model using preoperative and intraoperative data to forecast postoperative complications in GC patients undergoing radical gastrectomy, including those receiving neoadjuvant therapy. Key predictive factors included smoking, NRS2002score>3, reconstruction method, extent of resection, T-stage >1, operative time and neoadjuvant therapy combining immunotherapy or targeted therapy. Among the ten evaluated models, the XGBoost model demonstrated the highest AUC (0.788), exhibiting superior reliability and greater clinical decision-making benefit in predicting postoperative complications for GC patients. These findings highlight the significant potential of artificial intelligence in improving complication prediction and facilitating faster postoperative recovery in GC patients.

## Data Availability

The datasets generated and analyzed during the current study are not publicly available due the policy of the institution but are available from the authors on reasonable request. Requests to access the datasets should be directed to liruyinyixue@163.com.
